# Supplementary material for: Cytogenetic evidence supports Avena insularis being closely related to hexaploid oats
Source: PLoS One. 2021 Oct 15;16(10):e0257100. doi: 10.1371/journal.pone.0257100 (PMC8519437; doi:10.1371/journal.pone.0257100)
Supplement: S1 Table — (DOCX) [file pone.0257100.s001.docx]

**S1 Table. Occurrence and main distribution of SSRs in CCDD tetraploids: *A. insularis*, *A.magna* and *A. murphyi* and AACCDD hexaploids: *A. byzantina* and *A. sativa*.**

| **Oligonucleotide^a^** | **Genome A^b^** | **Genome C** | **Genome D** |
| --- | --- | --- | --- |
| (AC)_10_ | Distinctive signals in most chromosomes | Strong pericentromeric signals | Distinctive signals in most chromosomes |
| (CT)_10_ | Dispersed signals and distinct signals in two chromosomes of A*. sativa* | Dispersed and weak signals | Dispersed signals and distinct signals in one chromosome of *A. insularis* and hexaploids |
| (AAC)_5_ | Distinctive signals in three chromosomes | Distinctive signals in two chromosomes of *A. murphyi* and hexaploids | Distinctive signals in two chromosomes of hexaploids, *A. insularis* and A*. magna* and in four of *A. murphyi* |
| (AAG)_5_ | No signals | No signals | Distinctive signals in one chromosome of *A. insularis* and *A. magna* |
| (AAT)_5_ | No signals | No signals | No signals |
| (ACG)_5_ | Distinctive signals in one chromosome | No signals | Distinctive signals in one chromosome of *A. insularis* and three chromosomes in *A. magna* and *A. murphyi* |
| (ACT)_5_ | No signals | Distinctive signals in three chromosomes of both *A. insularis* and hexpaloids. In two chromosomes of *A .murphyi* | No signals |
| (AGG)_5_ | Dispersed signals | Dispersed signals | Dispersed signals |
| (ATC)_5_ | Distinctive signals in two chromosomes | Distinctive signals in one chromosome of *A. murphyi* | Distinctive signals in one chromosome of hexaploids, *A. insularis* and *A. murphyi* and in two chromosomes of *A. magna* |
| (CCG)_5_ | Dispersed signals | Dispersed signals | Dispersed signals |

^a^ Oligonucleotides tested in this work.

^b^ (AC)_10_ results for *A. magna*, *A. murphyi* and the hexaploids described in Fominaya et al. 2(017).

S1 Fig. FISH of mitotic metaphases of CCDD tetraploid species showing distribution of Am1 (red). Intergenomic translocations C/D are indicated by arrows. (a) *A. insularis*. (b) *A. maroccana*. (c) *A. murphyi*.
