# Supplementary figures and images for: Cytogenetic evidence supports Avena insularis being closely related to hexaploid oats
Source: PLoS One. 2021 Oct 15;16(10):e0257100. doi: 10.1371/journal.pone.0257100 (PMC8519437; doi:10.1371/journal.pone.0257100)

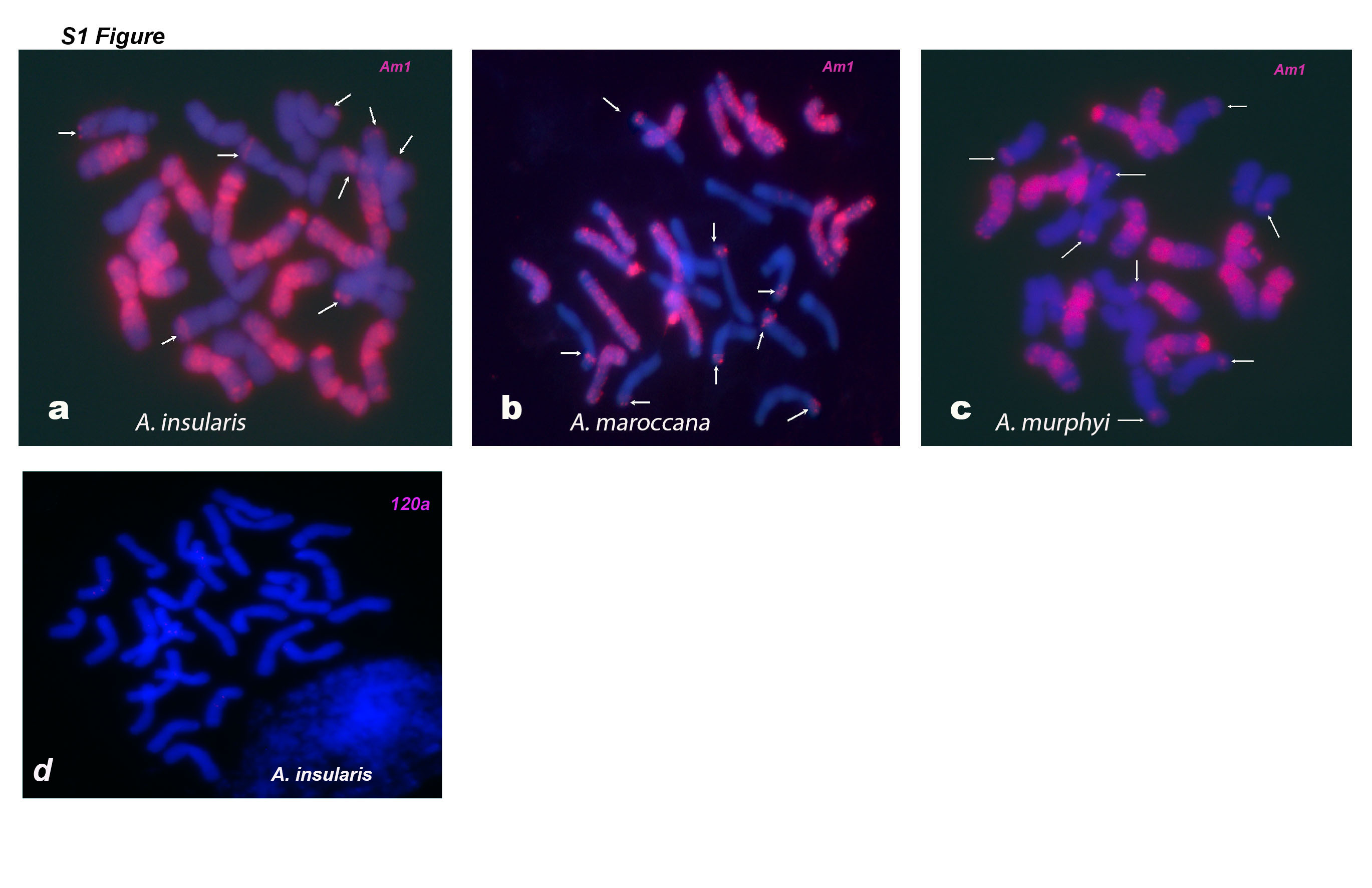

Supplement: S1 Fig — FISH of mitotic metaphases of CCDD tetraploid species showing the distribution of Am1 (red) (a–c) and 120a (red) (d). (a) A. insularis. (b) A. magna. (c) A. murphyi. (d) A. insularis. Arrows indicate C/D intergenomic translocations. (JPG) [file pone.0257100.s002.jpg]

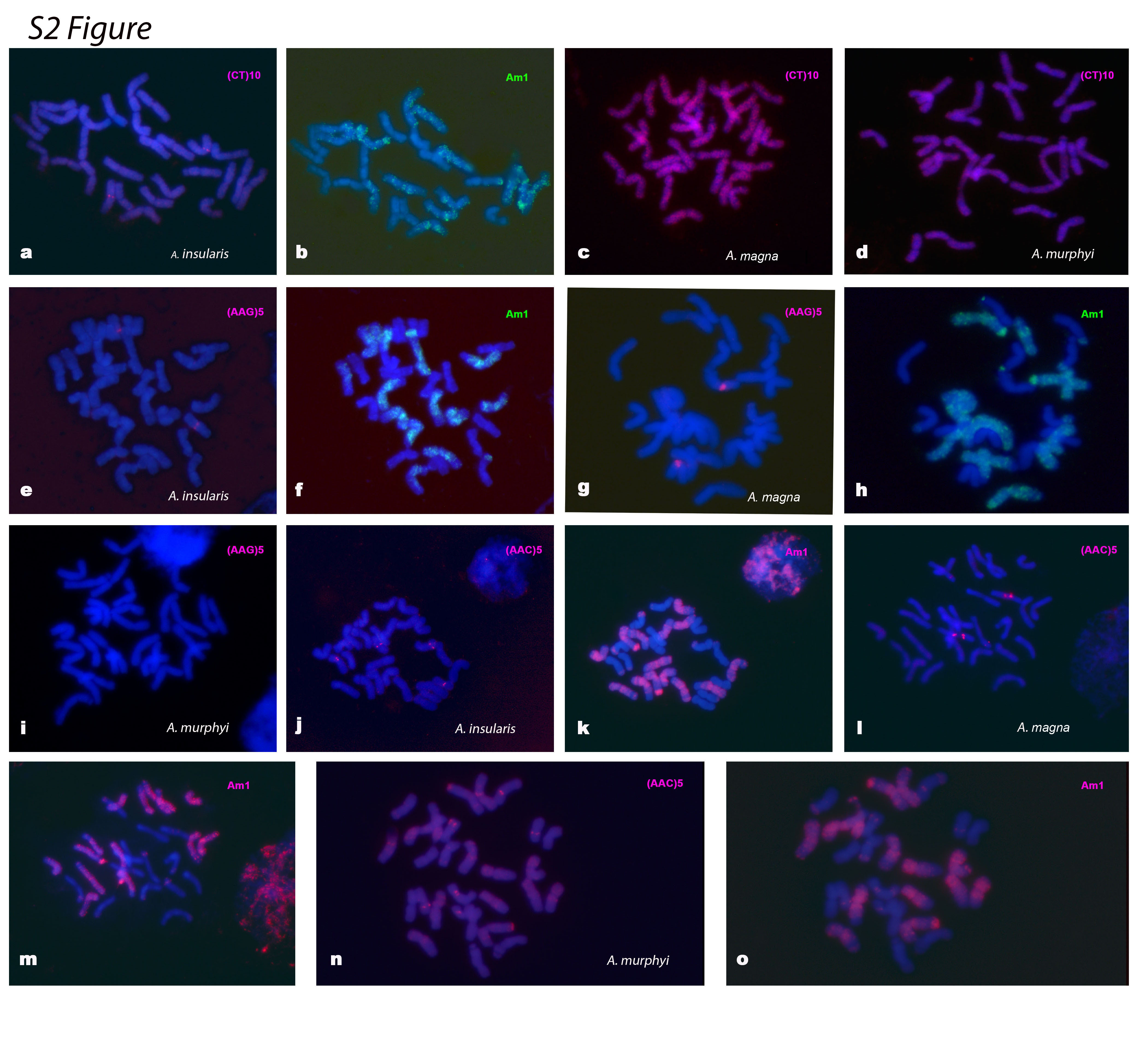

Supplement: S2 Fig — When positive signals for SSRs were observed, the same cells rehybridized with pAm1 are shown. (a and b) A. insularis. (c) A. magna. (d) A. murphyi. (e and f) A. insularis. (g and h) A. magna. (i) A. murphyi. (j and k) A. insularis. (l and m) A. magna. (n and o) A. murphyi. (JPG) [file pone.0257100.s003.jpg]

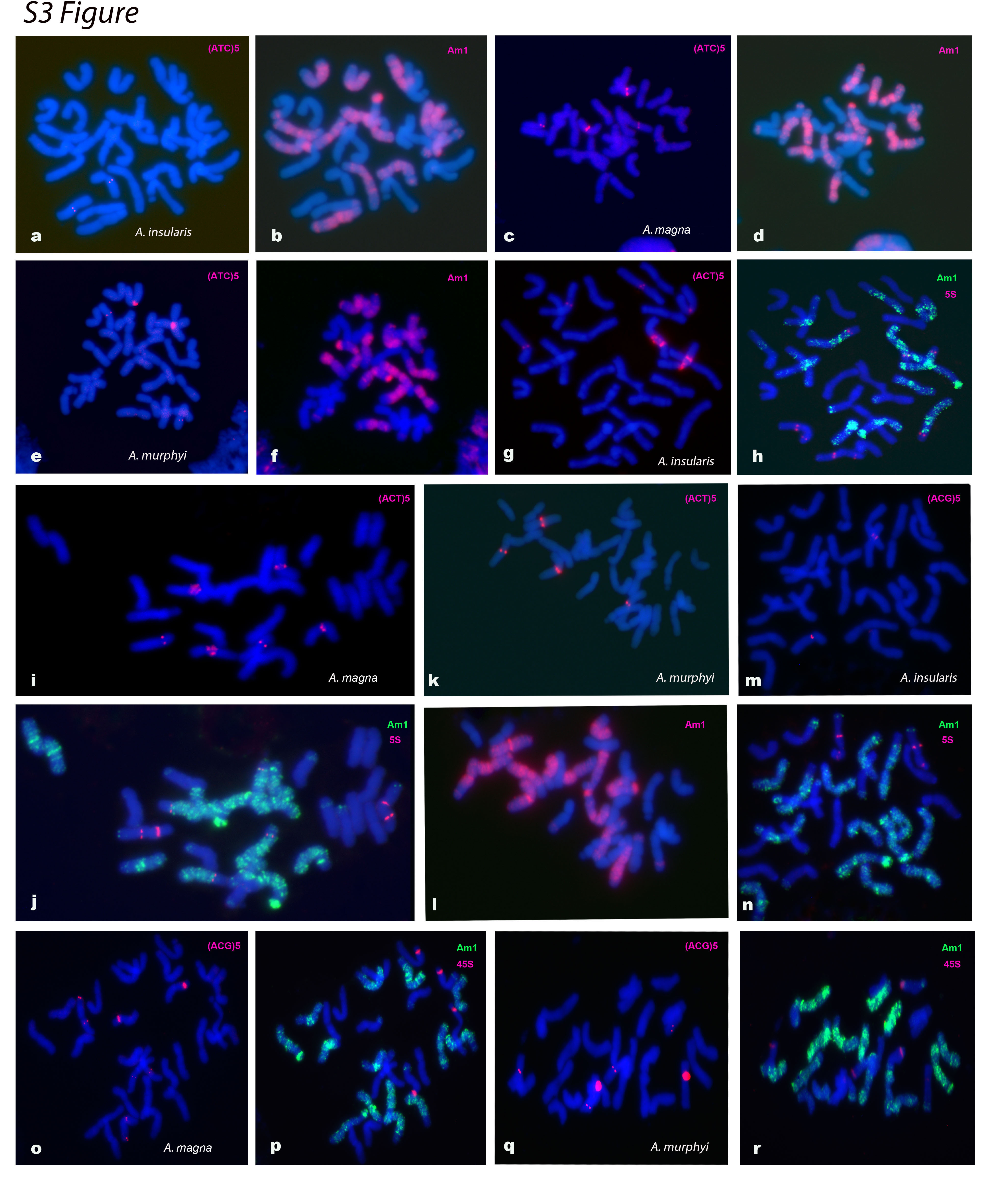

Supplement: S3 Fig — Same cells after rehybridization showing signals for Am1, 45S and 5S as indicated on the microphotographs. (a and b) A. insularis. (c and d) A. magna. (e and f) A. murphyi. (g and h) A. insularis. (i and j) A. magna. (k and l) A. murphyi. (m and n) A. insularis. (o and p) A. magna. (q and r) A. murphyi. (JPG) [file pone.0257100.s004.jpg]

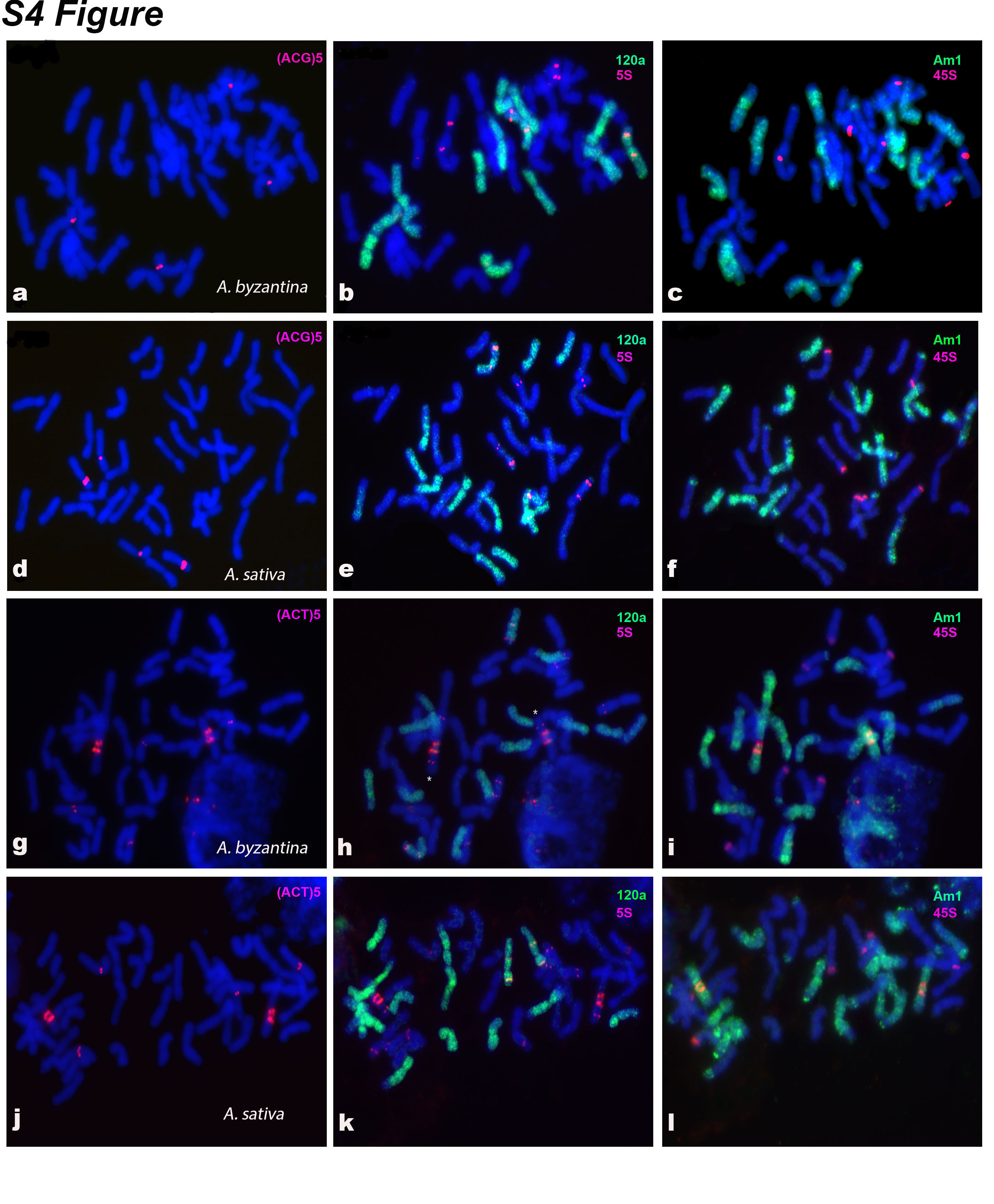

Supplement: S4 Fig — Same cells after rehybridization showing signals for Am1, 120a, 45S and 5S as indicated on the microphotographs. (a-c) A. byzantina. (d-f) A. sativa. (g-i) A. byzantina. (j-l) A. sativa. (JPG) [file pone.0257100.s005.jpg]

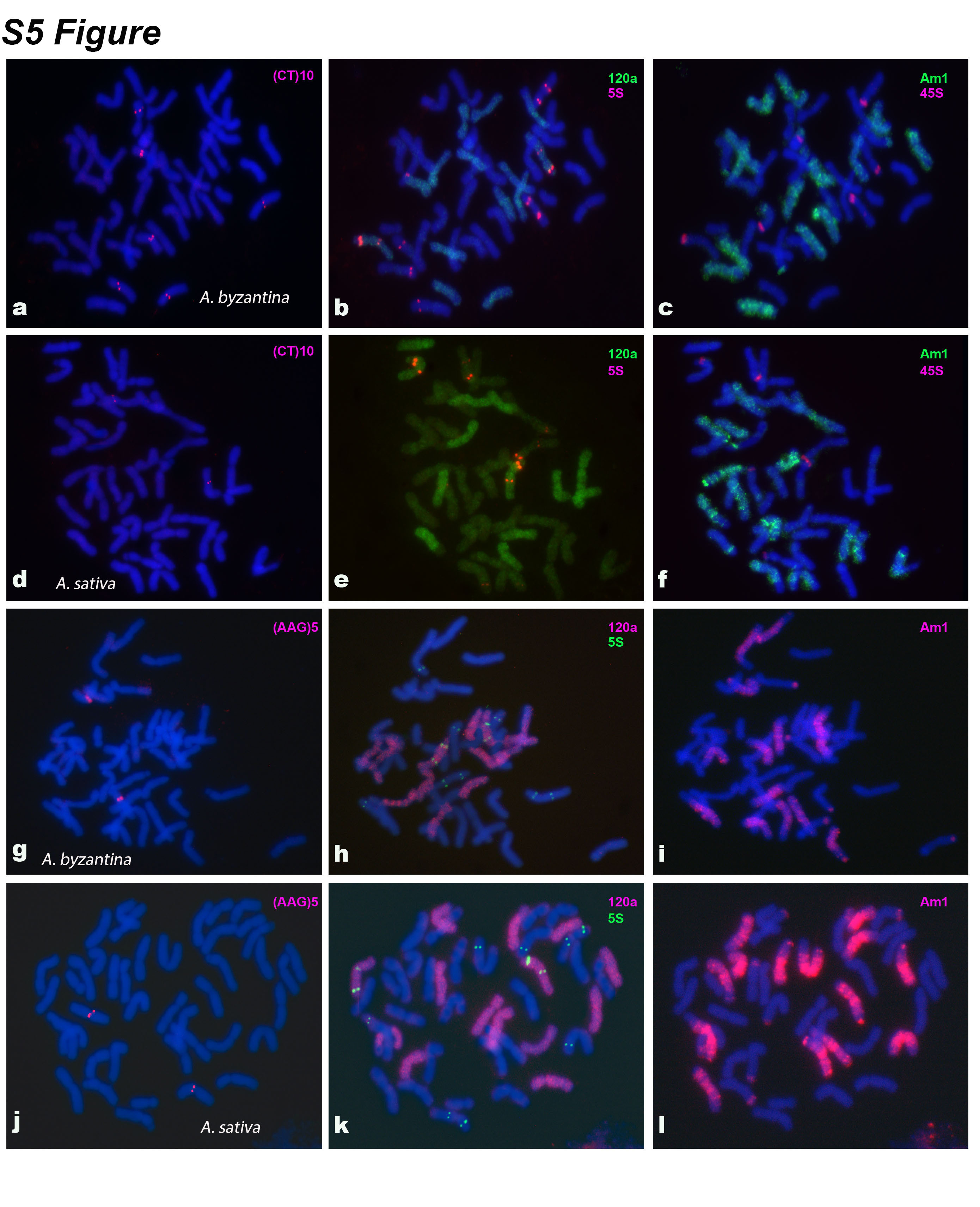

Supplement: S5 Fig — Same cells after rehybridization showing signals for Am1, 120a, 45S and 5S as indicated on the microphotographs. (a-c) A. byzantina. (d-f) A. sativa. (g-i) A. byzantina. (j-l) A. sativa. (JPG) [file pone.0257100.s006.jpg]

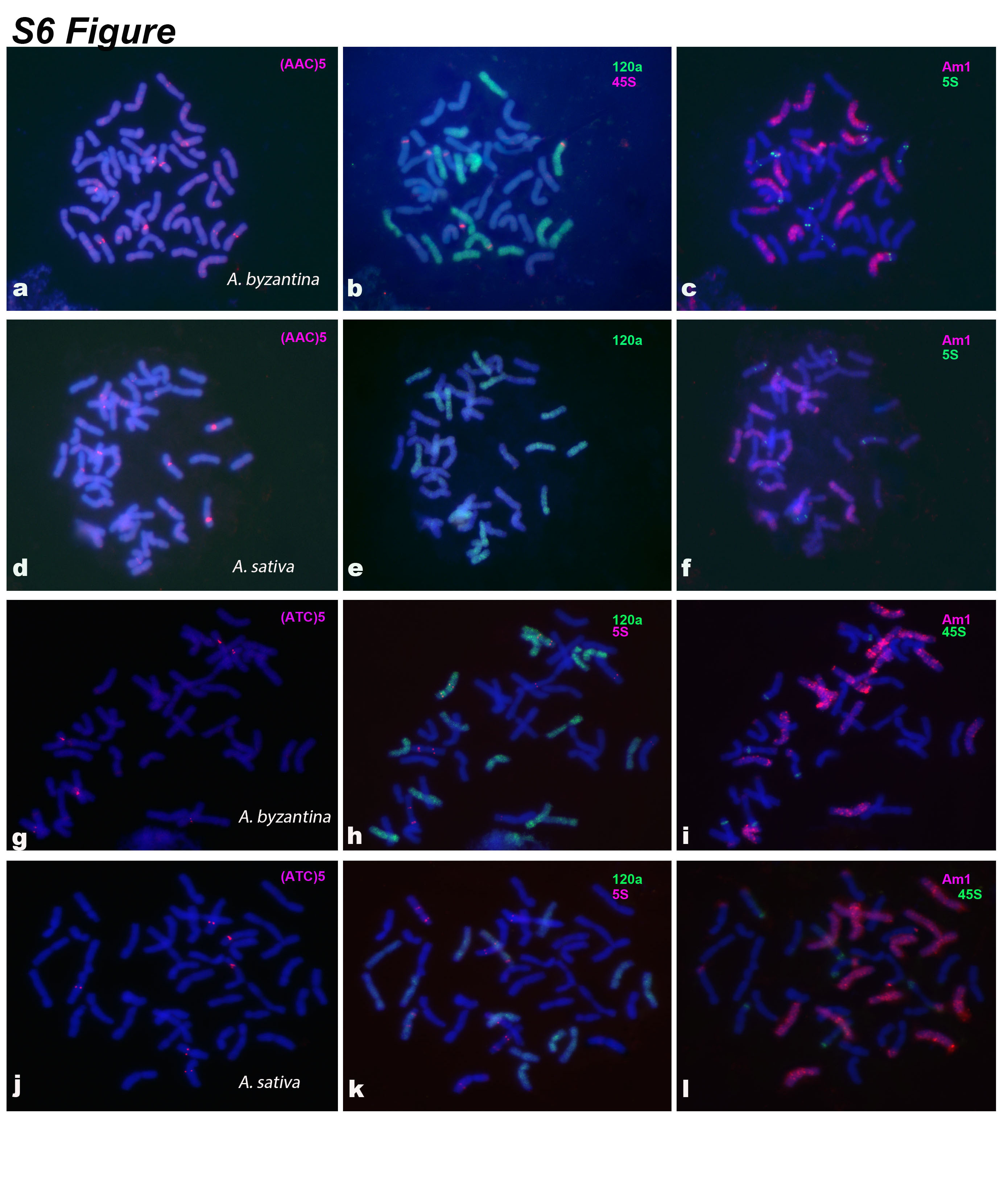

Supplement: S6 Fig — Same cells after rehybridization showing signals for Am1, 120a, 45S and 5S as indicated on the microphotographs. (ac) A. byzantina. (d-f) A. sativa. (g-i) A. byzantina. (j-l) A. sativa. (JPG) [file pone.0257100.s007.jpg]
